# The Effect of Graft Strength on Knee Laxity and Graft In-Situ Forces after Posterior Cruciate Ligament Reconstruction

**DOI:** 10.1371/journal.pone.0127293

**Published:** 2015-05-22

**Authors:** Yu-Shu Lai, Wen-Chuan Chen, Chang-Hung Huang, Cheng-Kung Cheng, Kam-Kong Chan, Ting-Kuo Chang

**Affiliations:** 1 Orthopaedic Device Research Center, National Yang-Ming University, Taipei, Taiwan; 2 Department of Medical Research, Mackay Memorial Hospital, New Taipei City, Taiwan; 3 Department of Biomedical Engineering, National Yang-Ming University, Taipei, Taiwan; 4 Department of Orthopaedics, Cardinal Tien Hospital, New Taipei City, Taiwan; 5 Department of Orthopedics, Mackay Memorial Hospital, Taipei, Taiwan; 6 Mackay Medical College, New Taipei City, Taiwan; Northwestern University Feinberg School of Medicine, UNITED STATES

## Abstract

Surgical reconstruction is generally recommended for posterior cruciate ligament (PCL) injuries; however, the use of grafts is still a controversial problem. In this study, a three-dimensional finite element model of the human tibiofemoral joint with articular cartilage layers, menisci, and four main ligaments was constructed to investigate the effects of graft strengths on knee kinematics and in-situ forces of PCL grafts. Nine different graft strengths with stiffness ranging from 0% (PCL rupture) to 200%, in increments of 25%, of an intact PCL’s strength were used to simulate the PCL reconstruction. A 100 N posterior tibial drawer load was applied to the knee joint at full extension. Results revealed that the maximum posterior translation of the PCL rupture model (0% stiffness) was 6.77 mm in the medial compartment, which resulted in tibial internal rotation of about 3.01°. After PCL reconstruction with any graft strength, the laxity of the medial tibial compartment was noticeably improved. Tibial translation and rotation were similar to the intact knee after PCL reconstruction with graft strengths ranging from 75% to 125% of an intact PCL. When the graft’s strength surpassed 150%, the medial tibia moved forward and external tibial rotation greatly increased. The in-situ forces generated in the PCL grafts ranged from 13.15 N to 75.82 N, depending on the stiffness. In conclusion, the strength of PCL grafts have has a noticeable effect on anterior-posterior translation of the medial tibial compartment and its in-situ force. Similar kinematic response may happen in the models when the PCL graft’s strength lies between 75% and 125% of an intact PCL.

## Introduction

Surgical reconstruction of damaged ligaments is a relatively new but rapidly developing option for the treatment of knee conditions. Most of these surgical treatments were originally developed for anterior cricuate ligament (ACL) reconstruction and then adapted to the posterior cruciate ligament (PCL). This is primarily due to the far greater incidence of injury to the ACL, but this does not withdraw from the severity of damage to the PCL. When the PCL is injured, progressive knee damage resulting from abnormal loading and joint laxity could lead to further knee pain, swelling, instability, and the onset of degenerative osteoarthrosis [1–4].

Limited medical data on PCL damage and a low number of studies performed to investigate such injuries greatly complicates the treatment of PCL ruptures [5]. Despite treatment of the PCL being a controversial issue, surgical reconstruction is recommended for patients with PCL-deficient knees [6–7]. However, the reconstruction of an isolated PCL tear can decrease tibial posterior laxity, but may not sufficiently restore the kinematics [8–10], which is believed to in connection with graft choice, graft fixation, tunnel creation, initial graft tension, etc. Because of insufficient clinical and biomechanical data, the selection of the appropriate graft for PCL reconstruction is still controversial. Some factors implicated in the failure of PCL reconstruction are similar to those identified in ACL failures, such as bone tunnel placement, pretensioning, size, strength and fixation method [11–14]. Weak graft anchorage during the remodeling phase has also been considered as another cause of failure [15]. Hence, information regarding graft strength and in-situ forces is important for a successful postoperative rehabilitation.

Harner et al. [16] used human cadaveric knees to investigate the in-situ forces in the PCL and the changes of knee kinematics under different load types. They found a strong relationship between the PCL in-situ force, load type, and tibial translation. Markolf et al. [15] used a load cell to measure the force generated in the PCL with a bone-patellar tendon-bone graft in human cadaveric knees. Their results indicated that the forces in a graft are slightly greater than in an intact PCL under a constant tibial loading. On the other hand, Lenschow et al. [17] reported a lower in-situ force in a hamstring tendon graft than an intact PCL. Thus, it implies that different graft strengths develop different in-situ forces, which may affect the initial graft fixation and post-operative knee kinematics. However, cadaveric studies, due to individual differences, are difficult to quantify with a specific graft and the effect of graft strengths on knee kinematics and PCL in-situ forces. To overcome these limitations, three-dimensional finite element models of the human knee joint have been used to analyze the biomechanical behavior [18].

Hence, the purpose of this study was to determine the effects of different PCL graft strengths on the knee kinematics and in situ forces of PCL grafts at knee extension.

## Materials and Methods

### Model Reconstruction

**Fig 1:**
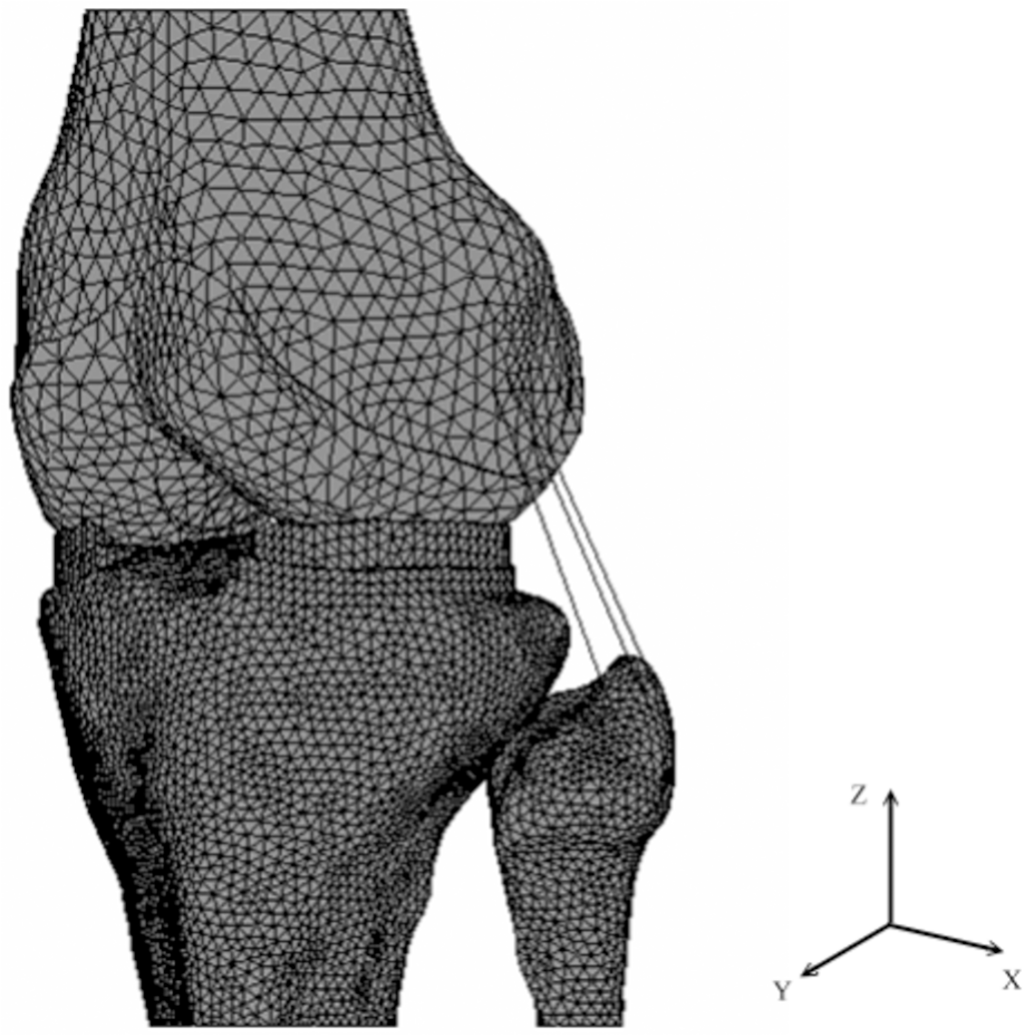
Finite element model of human knee including the femur, tibia, fibula, articular cartilage layers, menisci, and four main ligaments.

A three-dimensional finite element model was constructed from magnetic resonance imaging (MRI) scans of the left knee joint of a healthy male (with written consent to participate in this study. IRB approval by Mackay Memorial Hospital. Approval number: 12MMHIS209). The model meshes were generated using the MSC/PATRAN software (MacNiel-Schwindler Corp., Santa Ana, CA). Non-linear analysis and post-processing were performed with MSC/Mentat 2005 software (MacNiel-Schwindler Corp., Santa Ana, CA). This model consisted of three bony structures (femur, tibia and fibula), articular cartilage layers, menisci, and four main ligaments (collateral and cruciate ligaments). Non-linear cable elements were used to represent all ligaments, including four cable elements for both the ACL and PCL, and three cable elements for the LCL. The MCL was formed by eight cable elements, four were represented the proximal portion attaching the femur to the medial meniscus, and the other four represented the distal portion attaching the meniscus to the tibia (Fig 1) [18]. The global coordinate system defined the X, Y and Z axes as pointing in the directions of medial-lateral, posterior-anterior and proximal-distal respectively. Contact elements were assumed between the femoral cartilage and meniscus, the meniscus and tibial cartilage, and the femoral cartilage and tibial cartilage for both the lateral and medial areas. The model included six contact-surface pairs and the contact status was defined as ‘touching (sliding and rolling)’ in the software. Each contact surface was also modeled as frictionless [19]. The anterior and posterior horns of the menisci were fixed on the tibial cartilage to simulate an ‘unconstrained’ movement of the meniscus periphery. The gap between each contact element was adjusted to less than 0.15 mm to simulate the initial contact between femoral cartilage, tibial cartilage and meniscus [18]. Convergence tests was performed using six mesh densities in element sizes ranging from 6 mm^2^ down to 1 mm^2^ on the tibial cartilage and menisci. Under a compressive load of 890 N at knee extension, the boundary conditions on the top surface of the femur were set to be ‘fixed’, and the bottom surface of the tibia was set to be ‘constrained’ except for in proximal-distal (Z axis) translation. The calculated mean contact pressures on the medial and lateral compartments were used to check for model convergence. To take into account the elements’ aspect ratio and calculative efficiency of the computer, the solution was considered to converge with an element size of 2 mm^2^ on the meniscus and tibial cartilage and an element size of 4 mm^2^ on the other contact structures (Table 1). Thereby, the convergence model was composed of 110,294 tetrahedral elements (110,197 nodes) (Fig 1).

**Table 1:** The mean contact pressure in medial and lateral tibial cartilage and meniscus at 890 N for the six finite element mesh densities.

Element Size (mm)	Medial side		Lateral side	
	Mean Pressure (MPa)	Different Rate (%)*	Mean Pressure (MPa)	Different Rate (%)*
6 mm by 6 mm	2.01	36.73	1.52	24.59
5 mm by 5 mm	1.82	23.81	1.39	13.93
4 mm by 4 mm	1.66	12.93	1.32	8.20
3 mm by 3 mm	1.55	5.44	1.24	1.64
2 mm by 2 mm	1.49	1.36	1.22	0
1 mm by 1 mm	1.47	0	1.22	0

The differences in mean contact pressure were below 5% for the 2 mm^2^ mesh density

*Different Rate = [(The mean pressure of any one element size)-(The mean pressure of 1 mm by 1 mm)]/ (The mean pressure of 1 mm by 1 mm)

### Material Properties

The material behavior of the cortical bone, cancellous bone, cartilage and meniscus were all assumed homogeneous and linearly elastic. The elastic modulus and Poisson’s ratio were adopted from literature (Table 2) [18–19]. Both the ACL and PCL were assumed as two-bundle structures (ACL: anterior-medial bundle and posterior-lateral bundle; PCL: anterior-lateral bundle and posterior-medial bundle). The material properties of the two bundles of the ACL were identical, but each bundle of the PCL was given different material properties (Table 2) [18–21]. The stress-strain relationship of nonlinear ligament elements was described using the following equations:

$$\begin{array}{c} 0\;when\;\varepsilon <0\\ \sigma =\;\frac{1}{4}\kappa \frac{\varepsilon^{2}}{\varepsilon_{\iota }}\;when\;0\leq \varepsilon \leq 2\varepsilon_{\iota }\\ \;\kappa \left(\varepsilon -\varepsilon_{\iota }\right)\;when\;\varepsilon >2\varepsilon_{\iota } \end{array}$$

Where *σ* is stress, *κ* is the elastic modulus of ligaments, and *ε*_*ι*_ is assumed to be 0.03 as reference strain [22]. The aforementioned material properties were edited using Compaq Visual FORTRAN 6 (Compaq Computer Corp., CA) as a supplement to the MSC/Marc 2005 (MacNiel-Schwindler Corp., Santa Ana, CA) software. From the MRI data, the total cross-section areas were found to be 42, 62, 20, and 26 mm^2^ for the ACL, PCL, LCL, and MCL, respectively.

**Table 2:** The elastic modulus and Poisson’s ratio of cortical bone, cancellous bone, cartilage, meniscus and four ligaments.

	Elastic modulus	Poisson’s ratio
Cortical Bone	17 GPa	0.3
Cancellous Bone	350 MPa	0.25
Cartilage	12 MPa	0.45
Meniscus Matrix	10 MPa	0.45
Meniscus Horn	15 MPa	0.45
Anterior Cruciate Ligament	366 MPa	—
AL bundle of Posterior Cruciate Ligament	165 MPa	—
PM bundle of Posterior Cruciate Ligament	98 MPa	—
Medial Collateral Ligament	366 MPa	—
Lateral Collateral Ligament	366 MPa	—

### Boundary Conditions

In clinical settings, common grafts used for PCL reconstruction include hamstring tendons and bone-patellar tendon-bone (BPTB), and the elastic moduli of these grafts falls between 87 to 354 MPa, which is approximately 0.5 to 2 times that of an intact PCL [23]. Other detrimental factors such as insufficient graft strength during remodeling [15] may further reduce the graft’s effectiveness. Therefore, the strengths of different PCL grafts used in this study were defined from 0%-200% of an intact PCL’s elastic modulus, increasing in increments of 25%. A posterior drawer force of 100 N was applied to the tibial tubercle, and the boundary conditions on the top surface of the femur were set to be fixed in all directions, while the bottom surface of the tibia remained unconstrained except in flexion-extension [18]. These conditions assured a stable motion of the knee joint under a drawer force [18]. The kinematic response at knee extension was recorded, which included anterior-posterior tibial translation in medial and lateral compartments, internal-external rotation of the knee joint and changes of in-situ forces in PCL grafts with different strengths.

## Results

The results of validation, kinematic response and the graft’s in-situ forces in different models at full extension are described separately as follows.

### Model Validation

When the knee was at full extension and sustained a 100 N posterior drawer force, Fox et al. [24] used robotic technology to determine the forces in a human PCL and found that an intact PCL has a mean in-situ force of 35.6 N. This is very similar to our finding of 39.91 N in this study. Tibial posterior translation has been reported to range from 2 to 5 mm in an intact knee [18–19,25–26] and from 3 to 11 mm in a PCL-ruptured knee [18–19,27–29]. In our finite element analysis, the maximum tibial posterior translation was 3.60 mm in the intact knee (100%) and 6.77 mm in the knee with a ruptured PCL (0%). Previous studies have also reported noticeable posterior translation in medial tibial coupling with an abnormal internal tibial rotation in patients with a ruptured PCL [30,31]. Logan et al. [32] used open-access magnetic resonance imaging to investigate the tibiofemoral motion of PCL-deficient patients and indicated that the medial tibia was located 5 mm posterior to a normal knee at full extension. It indicated that the medial tibia shifted to a posterior position in PCL-ruptured knees. In our finial element analysis, a similar tendency was found for the PCL rupture model.

### Kinematics of the Knee Joint

**Fig 2:**
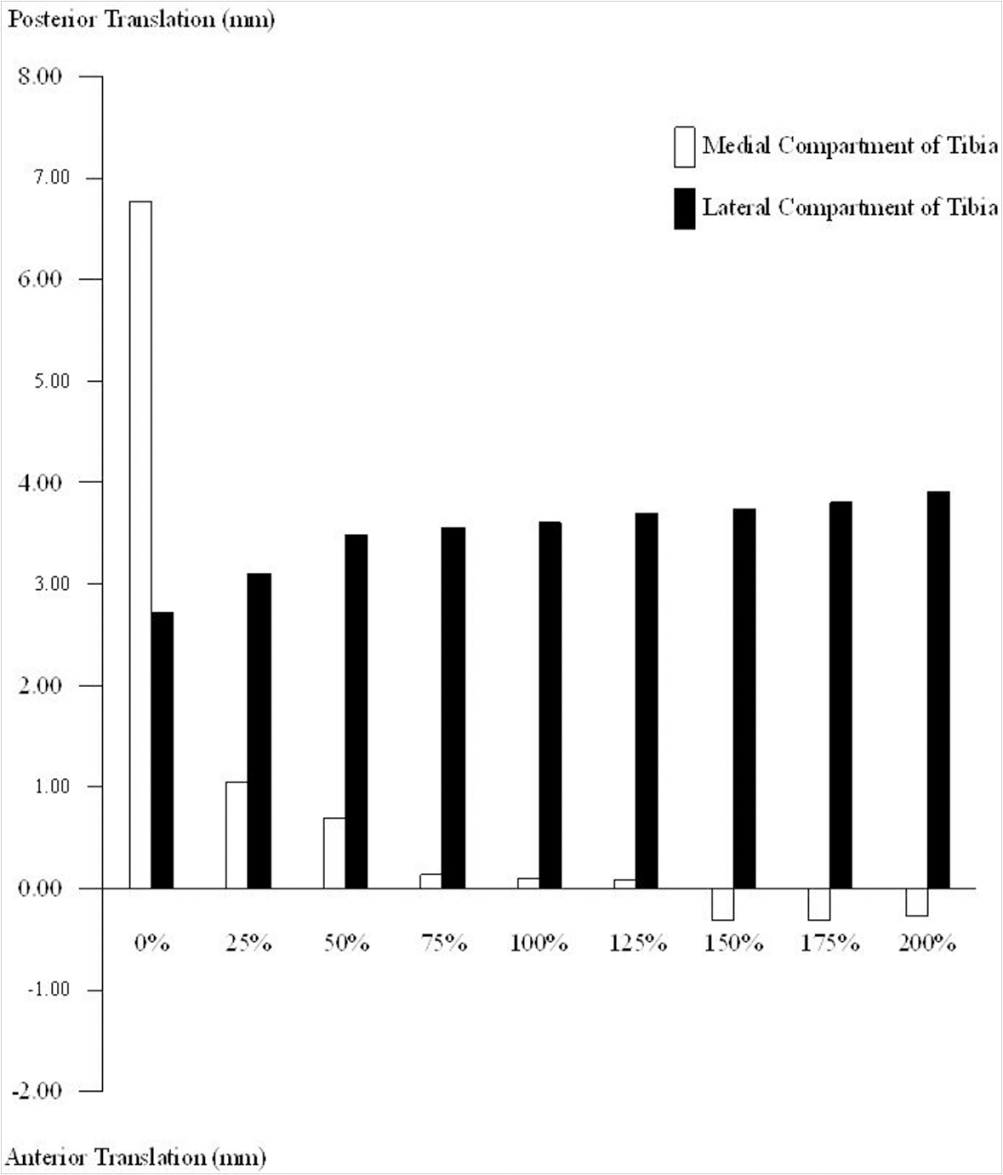
Anterior-posterior translations of medial and lateral tibial compartments in the reconstructed knee joint with different graft strengths. The anterior-posterior translations of the medial tibial compartment are noticeably affected by the graft strength.

**Fig 3:**
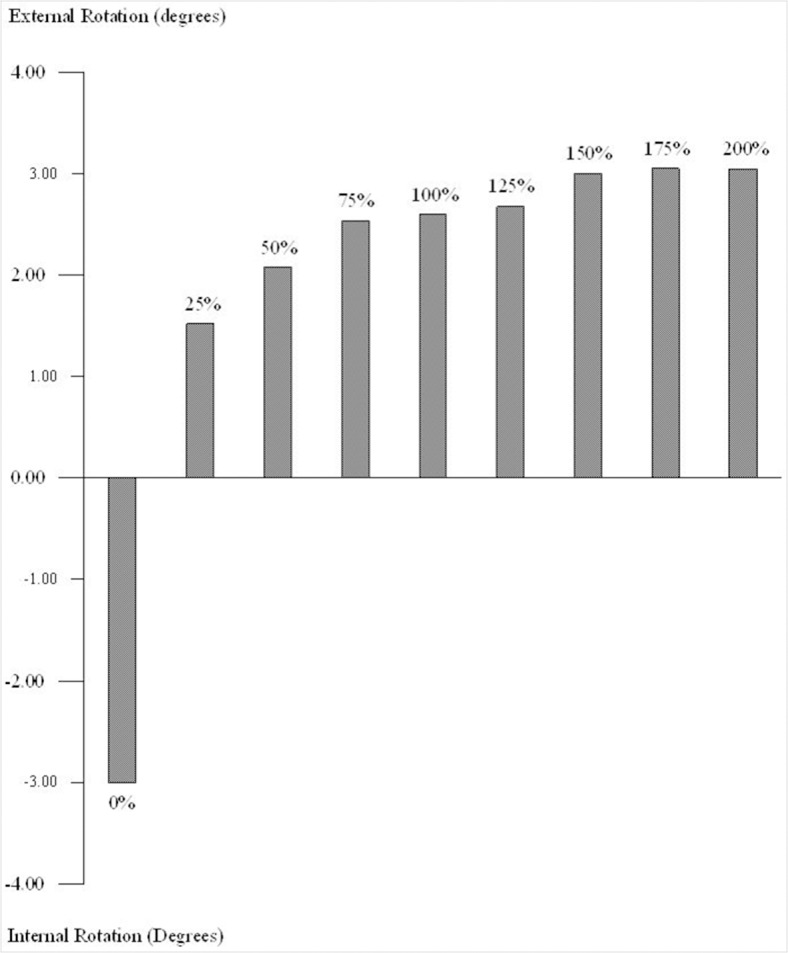
Tibial rotations in the reconstructed knee joint with different graft strengths. Internal tibial rotation occurred in the PCL fully-ruptured knee model. In all PCL reconstruction cases the tibia rotated externally.

Under the 100 N posterior drawer load, the normal PCL knee reached a maximum posterior translation of 3.60 mm in the lateral compartment (Fig 2) and a tibial external rotation of 2.60° (Fig 3). The maximum posterior translation of the knee with a ruptured PCL (0%) was 6.77 mm in the medial compartment (Fig 2), which resulted in tibial internal rotation of about 3.01° (Fig 3). The PCL rupture led to increase the sagittal laxity in the medial compartment of the tibia. After PCL reconstruction with any graft strength, the laxity of the medial tibial compartment was noticeably improved. When the 25% strength graft was used, the posterior translation of the medial tibia was only 1.05 mm, showing a decrease of about 5.62 mm in comparison to the PCL-ruptured knee, while external tibial rotation was 1.52° (Fig 3). The laxity of the medial tibia was reduced after PCL reconstruction, with increasing graft strengths offering further joint constraint. Tibial translation and rotation were similar to the intact knee after PCL reconstruction with graft strengths falling from 75% to 125% (Fig 2 and Fig 3). When the graft strength exceeded 150% of an intact PCL, the medial tibia moved forward; it translated anteriorly by 0.3 mm in the 150%, 175%, and 200% models. Also, when the strength surpassed 150%, external tibial rotation increased to 3.01°, 3.05° and 3.06° in the 150%, 175%, and 200% models, respectively (Fig 3).

### Graft In-Situ Forces

**Fig 4:**
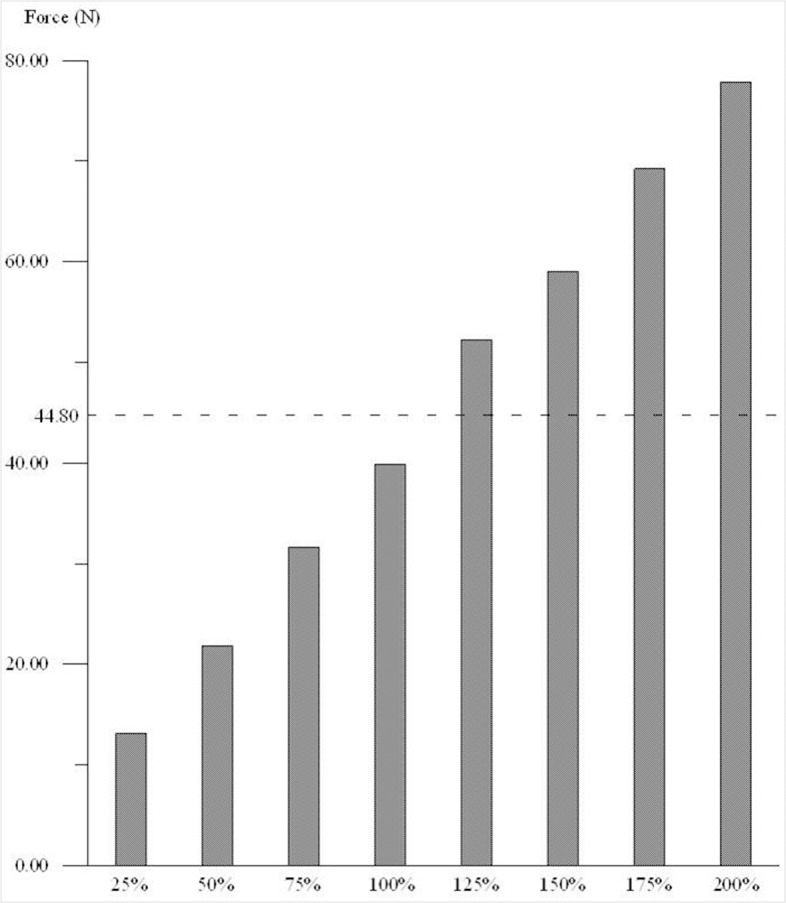
The in-situ forces in the grafts with different strengths under a 100 N posterior tibial force. The in-situ forces and graft strengths represented a proportional relationship.

With a 100 N posterior force applied to the tibia, a 39.91 N in-situ force was generated in the normal intact PCL at full extension. The force generated in the PCL grafts ranged from 13.15 N to 75.82 N; the in-situ force was related to the graft strength, with the lower strength grafts having a lower force. The force generated in the 25% strength graft was 13.15 N, representing on only 33% of an intact PCL (Fig 4). The in-situ forces for the 125%, 150%, 175%, and 200% strength graft models were 52.18 N, 59.01 N, 68.45 N, and 75.82 N, respectively (Fig 4).

## Discussion

The strength of PCL grafts is an important factor that affects the postoperative kinematics of the knee joint and the graft’s in-situ force. The aim of this study was to investigate the effects of different PCL graft strengths on knee kinematics and in-situ forces at knee full extension. To accomplish this study, a three-dimensional finite element model of a human knee joint was constructed with an atomic PCL.

There are a few limitations in this study that should be noted. First, the bone and soft tissues were assumed as homogeneous and isotropic, which is not representative of an anatomical knee. Hence, creep and stress relaxation could not be investigated in our model. Second, the special bundled structures of ligaments was not reconstructed accurately, but were simplified to be represented by cable elements. Although the interaction between different bundles of ligaments was not evaluated, it may still be important for knee stability [18]. Third, the joint capsule and other soft tissues around the knee joint were not reconstructed in this model and the effect of ligament position and ligament pre-straining were also not considered. Fourth, the graft strength of PCL reconstruction is affected by graft length, graft fixation and tunnel location in clinical. In this study, we ignored these variations and assumed they have equal initial graft length, fixation technique and tunnel location.

The strength of a PCL graft has a considerable effect on knee kinematics. The PCL grafts with 75% ~ 125% strength had a similar kinematic response to the intact PCL model. When the graft strength dropped below 50%, normal rotation of the tibia could not be restored. The graft with 25% strength caused a decrease in external tibial rotation and an increase in posterior translation of the medial tibia. However, if the graft strength exceeded 150%, the posterior translation of the medial compartment of the tibia became over-constrained and instead moved forward. In addition, the corresponding graft tensions were 48% greater than the tension in the intact PCL (Fig 4). Suggs et al. [33] found that using ACL grafts with higher stiffness than an intact ACL resulted in an over-constrained knee joint. Covey et al. [34] also indicated that PCL grafts with stronger mechanical properties caused tightening of the knee joint. Over-constraining the medial tibia would increase the tibiofemoral contact force, and the external rotation of tibia would further affect the “roll-back” mechanism. Thus the strength of PCL grafts not only affects the laxity of the medial tibia compartment but also plays an important role in controlling tibial rotation.

In response to a 100 N posterior tibial load, the in-situ force of the PCL grafts with different strengths ranged from 13.15 N to 75.82 N, becoming more severe with each successive increase in graft strength. The greater in-situ forces may cause higher stress concentrations at the graft fixation site and increase the risk of postoperative failure. Weiler et al. [35] used a sheep model to investigate the fixation strength of biodegradable interface screws in ACL reconstruction with tendon-to-bone grafts and reported graft failure at the screw insertion site. The mean failure forces were 44.8 N and 105.6 N at 6 and 9 weeks postoperatively. In our study, when the graft strength exceeded 125%, the in-situ forces were greater than 44.8 N, which would heighten the risk of graft fixation failure. However, the lower strength grafts produce a lower in-situ force to a posterior tibial load. Chen et al. [36] evaluated the fixation strength and failure types of three grafts (bone-patellar tendon-bone, hamstring tendon, and Achilles tendon) and the result indicated that the tendon-bone junction and the suture-tendon fixation site were the weakest points. The highest tensile stress values are often located at the graft’s femoral insertion zones. This is a common site of PCL rupture for a reconstructed PCL, as observed in some clinical cases [37]. Ramaniraka [37] et al. indicated that the tensile stresses in a high-stiffness PCL graft structure (double graft reconstruction) were greater than those of a single graft reconstructed PCL, and the high tensile stress within the graft may be the cause of fixation failure. Hence, grafts with high internal forces generated in response to muscle loading could accelerate graft failure. This study found that the strength of PCL grafts should lie between 75% and 125% of an intact PCL, which could decrease the risks of abnormal tibial rotation and early failure after PCL reconstruction.

Our results showed reported on changes of in-situ forces and knee joint kinematics due to different PCL graft strengths under the same loading conditions. This data may provide useful information on the biomechanical functions of the PCL, artificial tendon designs, and improve the selection of different PCL grafts.

## Conclusion

The structural strength of a graft plays an important role in determining the outcome of PCL reconstruction. This study showed that PCL graft strength noticeably affects the anterior-posterior translation of the medial tibial compartment but has little effect on the lateral tibial compartment. Similar kinematic response may happen in the models when the PCL grafts strength lies between 75% and 125% of an intact PCL. However, further study is needed to determine the effect of graft strength after PCL reconstruction at flexion position to be more relevant to PCL biomechanics.
